# BNC2 in Development and Disease: Regulatory Mechanisms and Translational Implications

**DOI:** 10.3390/molecules31122088

**Published:** 2026-06-14

**Authors:** Xianji Wei, Yuxiang Du, Xiaohua Liu, Lingli Zhang

**Affiliations:** 1 School of Athletic Performance, Shanghai University of Sport, Shanghai 200438, China; 2421852011@sus.edu.cn; 2 School of Exercise and Health, Shanghai University of Sport, Shanghai 200438, China; 2211517001@sus.edu.cn (Y.D.); 2511517008@sus.edu.cn (X.L.)

**Keywords:** Basonuclin 2, zinc finger transcription factor, non-coding regulatory variants, epigenetic regulation, chromatin remodeling, ovarian cancer susceptibility, fibrosis

## Abstract

Basonuclin 2 (BNC2) is a highly conserved cysteine–histidine (C2H2)-type zinc-finger nuclear regulatory protein characterized by three pairs of zinc-finger domains, a putative nuclear localization signal, a serine-rich region, broad tissue distribution, and remarkable transcript diversity generated through alternative promoter usage, alternative splicing, and polyadenylation. Increasing evidence from human genetics, animal models, functional genomics, and transcriptomic studies indicates that BNC2 links nuclear regulatory mechanisms to tissue-specific developmental and disease phenotypes. In the nervous system, BNC2-positive neuronal populations and BNC2-derived circular RNAs have been implicated in energy-balance circuits and neuroinflammatory regulation. In the skeletal system, BNC2 contributes to osteochondral development, periosteal stem-cell activation, chromatin remodeling, fracture repair, and genetic susceptibility to adolescent idiopathic scoliosis. BNC2 variants have also been associated with congenital lower urinary tract obstruction, whereas its expression and regulatory landscape are closely related to germ-cell development, epithelial ovarian cancer susceptibility, pigmentation traits, fibrosis, and several tumor contexts. Mechanistically, BNC2-associated phenotypes appear to involve cysteine–histidine zinc-finger-mediated transcriptional regulation, non-coding enhancer activity, epigenetic alterations, RNA-processing-associated nuclear functions, and chromatin-remodeling-dependent control of cell proliferation, differentiation, and stromal activation. This review integrates current evidence on the molecular architecture and regulatory functions of BNC2, critically discusses its context-dependent roles across development and disease, and highlights unresolved questions regarding isoform-specific activity, cell-type-specific regulation, downstream target networks, and clinical translation. A clearer understanding of these mechanisms may support the future evaluation of BNC2 as a biomarker, genetic susceptibility locus, molecular stratification factor, and potential therapeutic regulatory node.

## 1. Introduction

Basonuclin 2 (BNC2) is a member of the basonuclin protein family and a highly conserved cysteine–histidine (C2H2)-type zinc finger transcription factor [1]. The term “basonuclin” was originally introduced for a zinc-finger protein predominantly associated with the nuclei of basal keratinocytes, and BNC2 was subsequently identified as the second member of this basonuclin zinc-finger family [2,3]. Cysteine–histidine zinc finger transcription factors contain zinc-coordinating motifs stabilized by two cysteine and two histidine residues, allowing them to recognize specific DNA sequences and regulate gene-expression programs [4]. Compared with BNC1, BNC2 shows broader tissue distribution, greater transcript complexity, and more diverse disease associations, while retaining shared structural features such as three pairs of zinc-finger domains and a nuclear localization signal [5,6,7]. BNC2 is extensively expressed in tissues such as the testes, skin, kidneys, uterus, and intestines, indicating a potential role in the establishment and maintenance of tissue homeostasis [5].

Accumulating genetic, transcriptomic, and functional evidence has linked BNC2 to adolescent idiopathic scoliosis, congenital lower urinary tract obstruction, epithelial ovarian cancer susceptibility, male germ-cell development, and pigmentation [8,9,10,11,12]. More recent studies have further implicated BNC2 or BNC2-derived transcripts in hypothalamic neural circuit regulation, periosteal stem-cell-mediated fracture repair, fibrosis, and tumor-associated regulatory networks [13,14,15,16,17,18]. Rather than defining BNC2 solely as a developmental regulator, tumor suppressor, biomarker, or epigenetic factor, this review considers BNC2 as a context-dependent nuclear regulatory node. Its biological effects appear to be shaped by cell type, developmental stage, transcript diversity, non-coding regulatory variants, epigenetic state, chromatin organization, and disease microenvironment.

The regulatory complexity of BNC2 is supported by evidence from multiple molecular layers. Non-coding variants at or near the BNC2 locus can alter enhancer activity and transcription-factor binding, thereby influencing tissue-specific BNC2 expression and disease susceptibility [19]. DNA methylation studies have implicated BNC2-related epigenetic signatures in ovarian cancer risk [20], whereas circular RNAs derived from the BNC2 locus suggest an additional level of post-transcriptional and non-coding RNA-mediated regulation [21]. In addition, functional studies have linked BNC2 to chromatin-remodeling-dependent periosteal stem-cell activation during fracture repair and to transcriptional programs involved in fibrotic stromal activation [14,15]. These findings indicate that BNC2-associated diseases are not simply isolated clinical observations but may reflect the deployment of shared regulatory mechanisms in distinct cellular environments.

The current evidence on BNC2 spans several biological and disease contexts, including skeletal development and repair, lower urinary tract development, reproductive biology, pigmentation, neural regulation, fibrosis, and tumor-associated remodeling [8,9,10,11,12,13,14,15,19,20,21]. These fields collectively illustrate how BNC2 may connect non-coding regulatory variation, transcript diversity, chromatin regulation, and cell-state control to tissue-specific phenotypes [7,8,12,14,15,19]. However, the strength and nature of the evidence vary across systems. Some contexts are supported mainly by human genetic or regulatory-variant studies, whereas others rely more heavily on functional models, transcriptomic analyses, or disease-associated expression patterns.

Despite these advances, current evidence regarding BNC2 remains fragmented across organ systems and disease contexts. The relationships among its molecular architecture, regulatory mechanisms, and cross-disease functional patterns have not been systematically integrated. On this basis, this review first summarizes the molecular structure, transcriptional regulatory characteristics, and biological functions of BNC2. It then discusses BNC2-related evidence in the nervous, skeletal, urinary, reproductive, pigmentary, fibrotic, and tumor-associated contexts, with particular emphasis on how molecular regulatory mechanisms may explain its tissue-specific and disease-specific roles. Finally, we discuss unresolved mechanistic questions and the potential translational relevance of BNC2 in biomarker development, genetic risk assessment, molecular stratification, and therapeutic targeting.

### Literature Search and Selection Strategy

A focused narrative literature search was conducted using PubMed, Web of Science, Scopus, and Google Scholar. Search terms included “BNC2,” “Basonuclin 2,” “basonuclin-2,” “zinc finger transcription factor,” “non-coding variant,” “enhancer,” “epigenetic regulation,” “DNA methylation,” and “chromatin remodeling.” Original studies were prioritized when they provided direct evidence regarding BNC2 molecular features, transcriptional regulation, epigenetic mechanisms, animal models, human genetic associations, cell type-specific functions, or disease mechanisms. Relevant high-quality reviews were used only to support broader biological background. Recent studies were prioritized when they provided updated mechanistic, genetic, clinical, or translational evidence, whereas foundational earlier studies were retained when they represented the first description of BNC2 molecular features or experimentally validated biological functions. Studies were excluded if BNC2 was mentioned only incidentally, if the mechanistic or genetic relevance was unclear, or if the findings duplicated evidence from more comprehensive reports. As this is a narrative review rather than a systematic review or meta-analysis, no formal quantitative synthesis was performed.

## 2. Molecular Architecture of BNC2 and Its Functional Consequences

BNC2 is a nuclear regulatory factor with conserved zinc-finger architecture, nuclear localization, extensive transcript diversification, and nuclear speckle-associated properties [5,6,7]. Together with enhancer-dependent expression control and chromatin-remodeling-related activity, these features provide a molecular basis for its tissue-specific functions. This regulatory complexity may underlie the involvement of BNC2 in progenitor-cell proliferation, developmental patterning, RNA-processing-associated regulation, fibrotic activation, tumor-associated regulation, and genetic susceptibility to developmental disorders.

### 2.1. Conserved Zinc-Finger Architecture and Nuclear Regulatory Properties of BNC2

The initial identification of BNC2 provided the molecular foundation for subsequent studies of BNC2 biology. Romano et al. identified BNC2 as the second member of the basonuclin zinc-finger family of transcription factors and showed that mouse BNC2 contains three pairs of zinc fingers, a serine-rich region, and a nuclear localization signal. They further demonstrated that BNC2 transcripts are expressed in reproductive tissues, kidney, and skin, that recombinant BNC2 can bind a sequence within the ribosomal RNA gene promoter, and that full-length BNC2 localizes exclusively to the nucleus [3]. These findings established BNC2 as a DNA-binding nuclear regulatory protein and provided the basis for linking its structural features to transcriptional regulation and tissue-specific biological functions.

BNC2 is a member of the C2H2-type zinc finger protein family, and its amino acid sequence shows a remarkably high degree of conservation among vertebrates, indicating a stable and important role in nuclear gene regulation [5]. The human BNC2 gene is found on chromosome 9, has six exons, and produces a protein with about 1099 amino acid residues [6]. Structurally, BNC2 contains a serine-rich region, a putative nuclear localization signal, and three pairs of cysteine–histidine zinc-finger domains [5]. From the standpoint of structural biology, different types of zinc fingers are identified by their conserved locations and spacer lengths, whereas the stability of C2H2 zinc fingers is dependent on the coordination of zinc ions with cysteine and histidine residues [22]. These motifs commonly mediate sequence-selective DNA recognition, allowing C2H2 zinc finger transcription factors to engage specific genomic regions and regulate gene-expression programs [23,24]. High-affinity and high-specificity DNA binding is generally achieved by zinc-finger modules arranged in pairs or tandem arrays, while multiple spatially separated zinc-finger pairs can increase target-sequence specificity through combinatorial recognition [25].

These structural features are not merely descriptive protein motifs but are central to the presumed nuclear regulatory activity of BNC2. In BNC2, the presence of multiple spatially separated zinc-finger pairs suggests a potential capacity for combinatorial DNA recognition and cell-context-dependent target selection [1,3,5,7,26]. However, the precise genome-wide binding landscape and direct target genes of BNC2 remain incompletely defined. Existing functional studies nevertheless support its role as a nuclear regulatory protein. Basonuclin family proteins have been implicated in transcriptional control, and BNC2 differs from BNC1 in its broader tissue distribution, greater transcript complexity, and association with nuclear speckles [6]. Thus, BNC2 may act through both DNA-binding-dependent transcriptional regulation and RNA-processing-associated nuclear functions [27]. Its biological effects are therefore likely determined by the interaction among zinc-finger architecture, nuclear localization, isoform diversity, chromatin state, and cell type-specific regulatory context.

### 2.2. Transcriptional Regulatory Functions and Molecular Characteristics of BNC2

Within the BNC family, BNC1 and BNC2 share a paired zinc-finger organization, but their regulatory functions are not identical. Prior research has demonstrated that BNC1 can associate with rRNA gene promoters and enhance rRNA transcription [28,29]. Because the first zinc-finger pair of BNC2 is highly similar to that of BNC1, BNC2 has also been shown to bind rRNA promoter sequences in vitro, suggesting a potential relationship with ribosomal RNA transcriptional regulation [3]. However, subsequent functional evidence indicates that BNC2 should not be simply classified as a canonical RNA polymerase I transcription factor, and its broader nuclear functions may be more context-dependent [6]. The second and third zinc-finger pairs of BNC1 and BNC2 differ considerably, suggesting that the two proteins may have distinct target-gene preferences and biological functions [6].

In addition to its potential role in DNA-level transcriptional regulation, BNC2 exhibits molecular properties associated with nuclear speckles. Studies have shown that certain BNC2 isoforms localize to nuclear speckle regions and are associated with pre-mRNA processing, suggesting that BNC2 function may extend beyond transcriptional control to include RNA-processing-related nuclear functions [6]. BNC2 also exhibits marked transcript diversity. Through the combined use of six promoters, alternative splicing sites, and four polyadenylation sites, the human BNC2 gene has the theoretical potential to generate nearly 90,000 mRNA isoforms encoding more than 2000 possible protein products [7]. Compared with BNC1, BNC2 mRNA shows broader tissue distribution and is notably expressed in the testes, kidneys, uterus, and intestines [5]. BNC2 expression has also been detected in primary human keratinocytes and in HaCaT, HeLa, and HEK293 cells [3,7]. This broad expression pattern, together with its transcript complexity and nuclear speckle association, supports the view that BNC2 may exert cell type-dependent regulatory functions across different physiological contexts.

**Figure 1:**
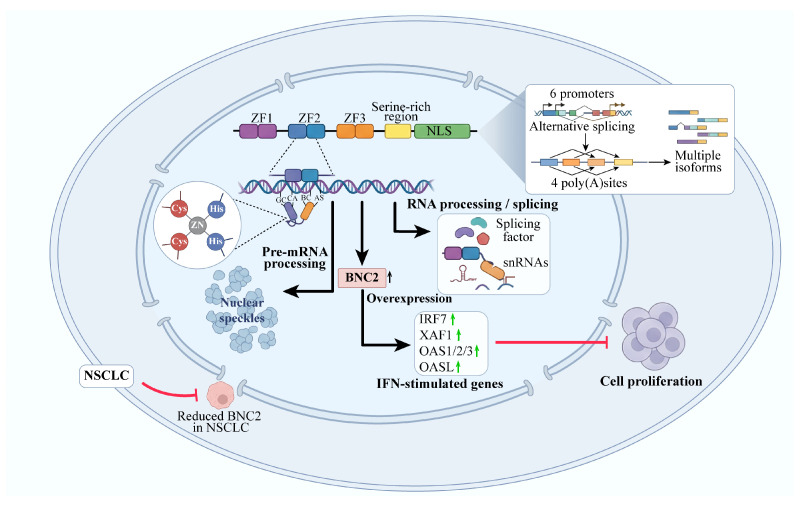
Molecular characteristics and regulatory functions of BNC2.

Functional evidence from a lung cancer model suggests that BNC2 can regulate disease-relevant transcriptional programs. Urgard et al. reported that BNC2 expression was reduced in non-small cell lung cancer tissues and A549 cells, whereas BNC2 overexpression in A549 cells upregulated interferon-stimulated genes, including IRF7, XAF1, OAS1, OAS2, OAS3, and OASL, and reduced cell proliferation [17]. These findings support a context-specific anti-proliferative and immune-related regulatory role of BNC2, rather than a universal tumor-suppressive function (Figure 1).

Therefore, BNC2 not only possesses the structural features of a zinc finger transcription factor but also exhibits nuclear speckle association, extensive transcript diversity, broad tissue expression, and context-dependent transcriptional outputs. These properties provide a molecular basis for understanding how BNC2 may contribute to distinct biological processes and disease phenotypes in a cell type-specific manner.

The schematic illustrates the conserved zinc-finger architecture, putative nuclear localization signal, serine-rich region, transcript diversification, nuclear speckle association, and RNA-processing-related features of BNC2. It also summarizes representative transcriptional outputs reported in a lung cancer model, in which BNC2 overexpression increased interferon-stimulated gene expression and reduced cell proliferation.

### 2.3. Molecular Features, Transcriptional Targets, and Cell-Type-Specific Functions of BNC2

BNC2-associated phenotypes span nervous, skeletal, urinary, reproductive, cutaneous, fibrotic, and tumor-related contexts. These diverse associations are supported by several recurring molecular features of BNC2, including zinc-finger-mediated transcriptional regulation, transcript heterogeneity, non-coding regulatory variation, chromatin remodeling, and cell type-specific gene-expression control. In lung cancer cells, BNC2 overexpression has been reported to induce interferon-related genes, including IRF7, XAF1, OAS1, OAS2, OAS3, and OASL, and to reduce cell proliferation, suggesting a context-specific role in anti-proliferative and immune-related transcriptional regulation [17]. The extensive use of alternative promoters, alternative splicing sites, and polyadenylation sites further suggests that different BNC2 isoforms may contribute to tissue- and cell-state-specific regulatory outputs [7]. In parallel, disease-associated non-coding variants can affect enhancer activity and transcription-factor binding, thereby altering BNC2 expression without changing the coding sequence. This mechanism has been reported in adolescent idiopathic scoliosis, pigmentation-associated traits, and ovarian cancer susceptibility [8,12,19]. BNC2 has also been linked to chromatin-remodeling-dependent regulation in periosteal stem cells during fracture repair and to TGF-β/Hippo-YAP1-related transcriptional programs during fibrotic activation [14,15].

The effects of BNC2 vary across cell types. In periosteal stem/progenitor cells, BNC2 contributes to chromatin accessibility at proliferation-related gene loci and supports injury-induced skeletal stem-cell expansion during fracture repair [14]. In fibrotic stromal cells, BNC2 promotes myofibroblastic activation and extracellular matrix remodeling, partly through matrisome- and collagen-associated transcriptional programs [15,16]. In hypothalamic BNC2-positive neurons, BNC2 marks a leptin-responsive neuronal population involved in the acute suppression of food intake [13]. In epithelial tumor contexts, reduced BNC2 expression or altered BNC2-related transcript regulation may be associated with impaired anti-proliferative or immune-related programs, whereas in stromal or fibrotic environments BNC2 may contribute to pathological matrix remodeling [15,16,17]. These findings indicate that BNC2-related biological outputs are strongly influenced by cell lineage, regulatory context, and disease microenvironment.

Direct BNC2-binding sites, primary target genes, and isoform-specific functions remain incompletely defined. Future studies integrating chromatin immunoprecipitation sequencing, assay for transposase-accessible chromatin sequencing, single-cell transcriptomics, spatial transcriptomics, and lineage-specific genetic models will be needed to clarify how BNC2-dependent regulatory programs differ across development, regeneration, fibrosis, and cancer.

## 3. Disease-Associated Functions of BNC2 from a Mechanistic Perspective

**Figure 2:**
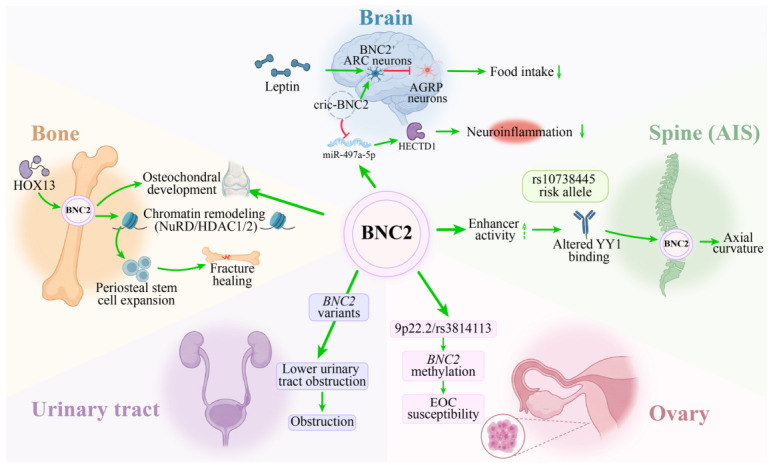
Multiorgan functions and disease-associated mechanisms of BNC2.

BNC2 has been implicated in multiple developmental and disease-related contexts, with recurring links to developmental gene-expression control, progenitor-cell proliferation and differentiation, enhancer-dependent tissue-specific expression, chromatin remodeling, tumor-cell behavior, and stromal activation [8,14,15,16,17,19]. The following sections summarize representative BNC2-associated disease contexts and the molecular mechanisms currently supported by genetic, transcriptomic, and functional evidence (Table 1, Figure 2).

The schematic illustrates representative BNC2-associated mechanisms in neural regulation, skeletal development and repair, lower urinary tract development, ovarian cancer susceptibility, and adolescent idiopathic scoliosis. These processes involve enhancer-dependent expression control, chromatin remodeling, non-coding RNA regulation, and cell type-specific transcriptional programs. Green arrows indicate promoting or activating effects, red blunt-ended lines indicate inhibitory regulation, and upward or downward arrows indicate increased or decreased changes, respectively.

### 3.1. BNC2 in Neural Circuit Regulation, Neuroinflammation, and Enteric Nervous System Biology

Although research on BNC2 in neurological disorders remains limited, current evidence suggests that BNC2-related signals may be involved in central metabolic circuits, neuroinflammatory regulation, and specific enteric neuronal populations. Tan et al. [13] identified a population of leptin-activated BNC2-positive neurons in the arcuate nucleus of the hypothalamus that rapidly suppress food intake by directly inhibiting AGRP neurons. Disruption of leptin receptor signaling impaired this regulatory circuit and was associated with hyperphagia and obesity. These findings indicate that BNC2 may play a role in regulating neuronal circuits connected with energy homeostasis and metabolism, although whether BNC2 itself is functionally required for this neuronal circuit remains to be clarified.

In addition to central metabolic regulation, circular RNAs derived from the BNC2 locus may participate in neuroinflammatory responses. Chen et al. [21] found that circ-Bnc2 expression was significantly downregulated in a lipopolysaccharide (LPS)-induced microglial inflammation model. Restoration of circ-Bnc2 suppressed inflammatory activation through the miR-497a-5p/HECTD1 axis and reduced apoptosis in neuron-like cells. This study suggests that BNC2-derived non-coding transcripts may contribute to neuroinflammatory regulation, but this evidence should be interpreted as transcript-specific rather than direct evidence for BNC2 protein function.

Single-cell and single-nucleus transcriptomic analyses have also detected BNC2-related expression signals in specific neuronal subpopulations of the enteric nervous system. Wright et al. [50] identified BNC2 expression in human and mouse enteric nervous system atlases, suggesting a potential association with enteric neuronal subtype identity or functional maintenance. However, direct pathogenic evidence linking BNC2 to enteric nervous system disorders remains lacking. Therefore, BNC2 should currently be considered a candidate marker or regulatory factor in enteric neuronal biology rather than an established driver of congenital neuroenteropathy or motility disorders.

Evidence connecting BNC2 to neurovascular disorders is even more limited. To date, signals near the BNC2 locus have been reported only in a genome-wide association analysis of sporadic cerebral arteriovenous malformations [51]. Because independent replication and functional validation are still lacking, this association should be interpreted cautiously.

Overall, current evidence suggests that BNC2-related neural functions are mainly supported by three lines of evidence: hypothalamic BNC2-positive neurons involved in energy-balance regulation, BNC2-derived circular RNAs, particularly circ-Bnc2, in neuroinflammatory models, and BNC2 expression in defined enteric neuronal populations. Further functional studies are needed to determine whether BNC2 acts as a causal regulator or primarily as a cell-state-associated marker in these neural contexts.

### 3.2. BNC2 in Osteochondral Development, Bone Repair, and Genetic Susceptibility to AIS

#### 3.2.1. BNC2 Participates in Osteochondral Development and Bone Repair

Evidence from developmental and genetic studies supports the expression and functional relevance of Bnc2 in bone-associated tissues. Vanhoutteghem et al. [32] detected strong Bnc2 expression signals in elastic and hyaline cartilage, the perichondrium, the nucleus pulposus of intervertebral disks, and perichondrial structures associated with long bones, suggesting that Bnc2 may contribute to the development and maintenance of osteochondral tissues. Furthermore, mice with systemic Bnc2 knockout exhibited a marked reduction in body size. Vanhoutteghem et al. [30] also found that Bnc2 deficiency leads to craniofacial structural abnormalities, a phenotype primarily attributed to impaired proliferation of embryonic craniofacial mesenchymal cells. Therefore, Bnc2 appears to be required for craniofacial mesenchymal cell expansion and normal formation of the palate and related osteochondral structures.

In addition to craniofacial development, Bnc2 has been implicated in the regulatory network governing distal limb development. By combining HOXA11/HOXA13 target-gene analysis with transcriptional regulatory evidence, Yamamoto et al. [31] showed that, during a specific developmental window in limb buds, HOX13 may transiently delay terminal chondrogenic differentiation by regulating a group of transcription factors, including Bnc2. This mechanism may provide the developmental timing and cellular expansion required for normal digit formation. Thus, Bnc2 may participate not only in osteochondral development, but also in the coordination of proliferation–differentiation transitions during skeletal patterning.

In recent years, the role of Bnc2 in bone injury repair has attracted increasing attention. Recent studies have shown that Bnc2 marks quiescent periosteal cells under steady-state conditions and is markedly upregulated in injury-responsive periosteal cells after fracture. Bnc2 deficiency in Prx1-lineage periosteal cells inhibits periosteal skeletal stem/progenitor cell proliferation and impairs fracture healing, whereas deletion in mature osteocytes or LepR-lineage bone marrow stromal cells does not produce comparable defects [14]. Mechanistically, Bnc2 deficiency reduces chromatin accessibility at promoters of proliferation-related genes and disrupts NuRD-associated chromatin remodeling. Partial rescue by HDAC1/2 inhibition further supports the role of Bnc2 in maintaining periosteal stem/progenitor cell activation and fracture repair through chromatin-dependent regulation.

#### 3.2.2. BNC2 and Genetic Susceptibility to AIS

Adolescent idiopathic scoliosis (AIS) is a common spinal deformity defined by a structural spinal curvature with a Cobb angle of 10° or more. It typically develops in otherwise healthy adolescents aged 10 to 18 years and shows marked sex dimorphism and genetic predisposition, with an estimated prevalence of approximately 2–3% [33]. Recent genetic studies have increasingly identified BNC2 as a candidate susceptibility locus for AIS [34,52]. Ogura et al. [8] identified the functional variant rs10738445 within an intronic region of BNC2 through cross-cohort association analysis. The risk allele was shown to enhance regulatory activity and alter binding of the transcription factor YY1, thereby influencing BNC2 expression. In zebrafish, BNC2 overexpression induced body-axis curvature during development, supporting a functional link between BNC2 dysregulation and scoliosis-related phenotypes [34].

Studies in Chinese populations have replicated associations between BNC2-related single-nucleotide polymorphisms and AIS susceptibility, with effect directions generally consistent with initial reports [34]. However, although the association between BNC2-related variants and AIS susceptibility appears relatively reproducible, their relationship with curve progression or scoliosis severity remains inconsistent across studies. This suggests that AIS onset and progression may be influenced by distinct combinations of genetic, developmental, biomechanical, and environmental factors [35].

Terhune et al. [53] systematically reviewed case–control genetic association studies of AIS and summarized evidence implicating multiple susceptibility loci, including variants within or near BNC2. This review-level evidence supports the relevance of BNC2 as an AIS-associated candidate locus but should be interpreted separately from original functional studies of specific regulatory variants. A recent genome-wide association study involving 119 AIS patients from 103 families further reported significant associations between variants within or near BNC2 and AIS. This study also placed the associated signals within broader biological pathways, including extracellular matrix remodeling and muscle contraction, suggesting that BNC2-related susceptibility may intersect with matrix regulation, biomechanical adaptation, and developmental processes during spinal growth [54].

Overall, current evidence supports BNC2 as a molecular link between osteochondral development, periosteal bone repair, and genetic susceptibility to AIS. However, its precise role in AIS pathogenesis remains incompletely defined. Future studies should clarify how BNC2-related non-coding variants affect tissue-specific expression, whether BNC2 regulates spinal growth through osteochondral, muscular, or matrix-associated pathways, and how these mechanisms differ between AIS onset and curve progression.

### 3.3. BNC2 Variants and Anatomical Lower Urinary Tract Obstruction

Lower urinary tract obstruction (LUTO) is a rare congenital condition characterized by obstruction of the bladder outflow tract, urinary retention, upper urinary tract dilatation, and secondary renal injury. Clinically, congenital LUTO can be broadly classified into anatomical obstruction, such as posterior urethral valves, urethral atresia, or strictures, and functional obstruction, which is usually related to abnormalities in neuromuscular regulation [55,56,57]. Among anatomical forms of LUTO, posterior urethral valves represent one of the most common causes in males, whereas urethral atresia can occur in both sexes but is less frequent [58]. Because anatomical LUTO is genetically heterogeneous, current evidence for BNC2 involvement should be interpreted by distinguishing human genetic observations from experimental model-based functional data.

#### 3.3.1. Human Genetic and Developmental Evidence

Human evidence supporting a role for BNC2 in lower urinary tract development comes from both developmental expression studies and rare-variant analyses. Bhoj et al. reported a human balanced translocation disrupting BNC2 and showed that BNC2 is highly expressed in fetal periurethral tissues, supporting a role in distal urethral development [37]. Mouse gene inactivation in the same study further produced distal urethral defects, providing mammalian developmental evidence that complements the human observation. Kolvenbach et al. subsequently identified rare BNC2 variants in families affected by congenital lower urinary tract obstruction [9]. Specifically, they reported a truncating BNC2 variant in a family with four affected individuals and a missense variant in another family with two affected members. Resequencing of BNC2 transcripts in additional patients with lower urinary tract obstruction identified one potentially pathogenic variant and two variants of uncertain clinical significance in individuals with urethral stricture or posterior urethral valves. These findings suggest that BNC2 may contribute to anatomical LUTO in rare familial cases, but the available human evidence remains limited and is not yet supported by large population-level association studies.

#### 3.3.2. Zebrafish Functional Modeling

Zebrafish studies provide functional support for the relevance of BNC2 in lower urinary tract development. In zebrafish embryos, bnc2 expression has been detected in the pronephric duct and cloacal region, which are considered developmental analogs of the mammalian lower urinary tract. Experimental knockdown of bnc2 induces distal pronephric outlet obstruction and cloacal dilatation, thereby phenocopying key aspects of human congenital LUTO [9,38]. The zebrafish model is particularly useful for this question because its transparent embryos, rapid development, and suitability for genetic manipulation allow direct visualization of distal urinary tract malformations [38]. More recent zebrafish-based studies have also shown that morpholino knockdown or CRISPR/Cas F0 mosaic disruption of candidate genes can model developmental lower urinary tract malformations [38]. Thus, zebrafish evidence supports a functional requirement for bnc2 in distal urinary outlet development, but it should be interpreted as cross-species functional validation rather than direct human genetic evidence.

Taken together, the current evidence linking BNC2 to congenital lower urinary tract obstruction consists of two complementary layers: rare-variant and developmental-expression evidence from human families and mammalian tissues, and functional support from zebrafish developmental models. However, the number of reported human families remains limited, and the contribution of BNC2 variants to sporadic LUTO, posterior urethral valves, or broader congenital urinary tract malformations requires validation in larger independent cohorts. Future studies should also distinguish coding variants from structural or non-coding regulatory variants and clarify whether BNC2 acts through urethral mesenchymal development, epithelial differentiation, or broader cloacal patterning programs.

### 3.4. BNC2 in Reproductive Biology and Disease

#### 3.4.1. BNC2 in Epithelial Ovarian Cancer Susceptibility and Biology

Epithelial ovarian cancer (EOC) comprises biologically heterogeneous tumors with distinct cells of origin, molecular features, and genetic risk architectures. High-grade serous ovarian carcinoma (HGSOC), the most lethal subtype, is strongly linked to fallopian tube secretory epithelial precursors, although ovarian surface epithelium and endometriosis-associated epithelial lesions may contribute to other ovarian cancer subtypes [40,41]. In this context, BNC2 has attracted attention because genetic, regulatory, functional, and epigenetic studies have repeatedly linked the 9p22.2/BNC2 region to ovarian cancer susceptibility and tumor biology [42].

##### GWAS Evidence and the 9p22.2/BNC2 Susceptibility Locus

Genome-wide association studies first identified the 9p22.2 region as an ovarian cancer susceptibility locus, with rs3814113 emerging as one of the representative variants associated with EOC risk. The minor allele of rs3814113 has been associated with reduced ovarian cancer risk, particularly for serous ovarian cancer, suggesting that inherited regulatory variation at this locus may influence disease susceptibility [10]. Subsequent studies further showed that the 9p22.2 region can act as a risk-modifying locus in carriers of pathogenic BRCA1 or BRCA2 variants, indicating that BNC2-associated regulatory variation may contribute to ovarian cancer risk in both the general population and genetically predisposed groups [42,43]. These findings support BNC2 as a susceptibility-associated locus, although GWAS evidence alone does not establish the precise causal variant, disease-relevant cell type, or downstream target network.

##### Enhancer–Promoter Regulation at the BNC2 Locus

Fine-mapping and functional annotation studies have provided a more mechanistic interpretation of the 9p22.2 susceptibility signal. The BNC2 region contains multiple regulatory elements, including enhancer regions and promoter-interacting sequences, that may influence BNC2 transcription and downstream gene networks. Buckley et al. characterized the regulatory landscape of the 9p22.2 locus and proposed that ovarian cancer susceptibility may be mediated through coordinated effects on BNC2 expression and related regulatory networks [19]. This enhancer–promoter model explains how non-coding susceptibility variants may influence ovarian cancer risk without altering the BNC2 coding sequence.

##### BNC2 as a Putative Tumor Suppressor in High-Grade Serous Ovarian Carcinoma

Beyond inherited susceptibility, tumor biology studies suggest that BNC2 may have tumor-suppressive properties in specific HGSOC contexts. Cesaratto et al. reported reduced BNC2 expression in high-grade serous ovarian carcinoma and showed that BNC2 affects cellular survival under oxidative stress [18]. These findings suggest that loss or reduction in BNC2 activity may impair stress-response programs and contribute to tumor-cell survival in specific ovarian cancer contexts. However, BNC2 should not be interpreted as a universal tumor suppressor across all cancers, because its function appears to depend on tumor subtype, cellular compartment, stromal context, and disease stage.

##### Epigenetic Markers and Translational Implications

Epigenetic evidence further supports the relevance of BNC2 in ovarian cancer susceptibility. Regional blood-based DNA methylation analyses adjusted for blood cell composition have implicated BNC2-related methylation patterns in ovarian cancer risk, suggesting that BNC2 may also be involved in epigenetic susceptibility signatures [20]. These findings raise the possibility that BNC2-related genetic and epigenetic markers could contribute to future risk stratification or biomarker development. Nevertheless, their translational application remains preliminary. Further studies are needed to define the causal regulatory variants, disease-relevant cell types, subtype-specific effects, direct transcriptional targets, and clinical utility of BNC2-related genetic or epigenetic markers in ovarian cancer.

#### 3.4.2. BNC2 in Male Germ Cell Development

During mammalian germ cell development, the timing of meiotic entry differs markedly between male and female germ cells. Unlike female germ cells, which initiate meiosis shortly after entering the fetal gonads, male germ cells remain largely undifferentiated during fetal development and gradually enter meiosis and complete spermatogenesis after birth [39]. In the male mouse germline, Bnc1 and Bnc2 can be detected in fetal spermatogonia and undifferentiated spermatogonia, but their expression rapidly declines once spermatogonia enter the differentiation stage [7,11,32]. Subsequent studies have shown that BNC2 is enriched in primitive germ cells and undifferentiated spermatogonia and exhibits multiple testis-associated splice variants, suggesting a specialized role in early male germ cell development [32]. In contrast, during oogenesis, BNC family proteins tend to accumulate in mature oocytes, indicating that their temporal functions may differ between male and female germ cells [32].

At the mitotic level, BNC2 appears to be associated with the maintenance of undifferentiated male germ cell populations. Studies have shown that BNC2 is preferentially expressed in primitive testicular germ cells, particularly fetal and adult undifferentiated spermatogonia [11,32]. This expression pattern suggests that BNC2 may contribute to the preservation of the undifferentiated spermatogonial pool and the regulation of early germ cell developmental programs. At the meiotic level, available evidence suggests that BNC2 may help prevent premature meiotic entry while supporting subsequent meiotic progression and stem-cell pool homeostasis [11]. BNC2 may also influence meiosis-associated processes, including chromosome pairing, recombination, or synapsis, although the direct molecular targets remain incompletely defined. Overall, current evidence supports BNC2 as a regulator of early male germ cell development, particularly in the balance between undifferentiated spermatogonial maintenance and meiotic progression.

### 3.5. Non-Coding Variants, Pigmentation, Cancer, and Fibrosis

#### 3.5.1. BNC2-Associated Non-Coding Variants and Pigmentation Phenotypes

Human pigmentation is a complex quantitative trait shaped by multiple genetic loci, environmental exposure, and phenotype definition [59]. In this context, BNC2 should be considered a pigmentation-associated regulatory gene rather than a universal determinant of all pigmentation phenotypes. Genome-wide association studies and candidate-gene analyses have linked variants within or near the BNC2 locus to skin pigmentation traits in European and Asian populations [12,44,47]. Functional analyses further refined this association to the non-coding enhancer variant rs12350739, showing that conserved sequences adjacent to this locus exhibit enhancer activity and regulate BNC2 transcription in melanocytes in an allele-dependent manner [12]. This provides direct evidence that non-coding regulatory variants can influence pigmentation-related phenotypes by modulating BNC2 expression rather than altering the coding sequence.

Quantitative phenotyping has been particularly important for clarifying the contribution of BNC2 to pigmentation. A genome-wide association study in a Korean female cohort used CIELAB color indices to assess facial skin tone and identified an association between the BNC2 locus variant rs16935073 and facial skin color parameters [47]. Candidate-gene studies in European populations also associated BNC2 with continuous skin color variation, especially skin color saturation [44]. These findings suggest that BNC2-associated effects may be more readily detected using quantitative colorimetric traits than traditional categorical pigmentation phenotypes.

In addition to baseline skin color, genome-wide association studies of facial pigmented spots have identified variants in or near several pigmentation-related genes, including IRF4, MC1R, RALY/ASIP, and BNC2 [60]. This indicates that BNC2-associated variants may also contribute to acquired facial pigmentation phenotypes, although the strength of this association may vary by population background, phenotype definition, and measurement method. Cross-species evidence further supports a functional link between BNC2 and pigmentation biology, as bnc2 disruption in zebrafish affects adult pigment pattern formation and chromatophore organization [61]. Overall, current evidence supports BNC2 as a non-coding regulatory locus involved in pigmentation-related traits, but its contribution should be interpreted within the broader polygenic architecture of human pigmentation.

#### 3.5.2. Functional Roles of BNC2 in Fibrosis and Multiple Cancer Contexts

Evidence from fibrosis and cancer studies indicates that BNC2 has context-dependent functions that may differ between epithelial tumor cells, stromal cells, and non-coding RNA-mediated regulatory networks. In fibrotic models, Bobowski-Gerard et al. showed that BNC2 is markedly upregulated during fibrotic activation and promotes fibrotic progression through TGF-β- and Hippo/YAP1-related signaling [15]. Genetic loss of Bnc2 in a mouse model of liver fibrosis reduced collagen deposition and attenuated fibrosis severity. Recent work by Orang et al. [16] further expanded this concept by showing that BNC2 regulates extracellular matrix production and degradation and influences cell migration, fibroblast-associated matrix remodeling, and cancer-cell behavior. These findings suggest that BNC2 may promote pathological matrix remodeling in stromal or mesenchymal contexts, providing a mechanistic explanation for why BNC2-related functions can differ across disease settings.

In hepatocellular carcinoma, available evidence suggests that BNC1 and BNC2 show aberrant expression in tumor tissues and cell lines and may be linked to tumor-associated regulatory mechanisms [62]. Elevated promoter methylation of BNC2 appears to be relatively uncommon in hepatocellular carcinoma, whereas reduced expression may be more closely related to structural alterations such as gene deletion [62]. Thus, BNC2 downregulation in hepatocellular carcinoma may reflect multiple mechanisms, including genomic loss and epigenetic dysregulation, rather than a single uniform silencing mechanism.

In gastric cancer, genomic and epigenomic studies have also implicated BNC2 in specific tumor subtypes. Comparative mutation profiling of gastric adenocarcinoma showed that recurrent loss-of-function variants in genes including ABCA10, BNC2, CDH1, CTNNB1, and SMAD4 are enriched in the B1 epigenomic subtype [63]. In addition, computational analyses of early-stage gastric cancer identified a recurrence-associated gene signature that includes BNC2 in the TCGA stage I–II cohort [64]. This signature was associated with poor prognosis despite relative genomic quiescence and low mutational burden. These findings suggest that BNC2 may be incorporated into molecular stratification frameworks in gastric cancer, although functional validation remains necessary.

BNC2 has also been included in tumor microenvironment-related signatures. In bladder cancer, a fibroblast-associated expression signature containing BNC2, LAMA2, MFAP5, NID1, and OLFML1 was reported to support risk classification based on TCGA and GEO datasets [65]. This association suggests a possible link between BNC2 and fibroblast-related tumor microenvironment remodeling, but the underlying mechanism and its relationship with treatment response require further experimental validation.

The role of BNC2-related transcripts is not limited to linear mRNA. Recent studies have shown that circBNC2 is downregulated in prostate cancer, castration-resistant prostate cancer, and neuroendocrine prostate cancer tissues. Mechanistically, circBNC2 was reported to promote ferroptosis through the circBNC2/miR-4298/ACSL6 axis, thereby inhibiting tumor cell proliferation, migration, and invasion [49]. A nanotherapeutic strategy combining circBNC2 delivery with docetaxel further showed antitumor potential in this context. However, these findings should be interpreted as evidence for BNC2-derived circular RNA function rather than direct evidence for BNC2 protein activity.

Overall, current evidence indicates that BNC2-related mechanisms are highly context-dependent across fibrosis and cancer. In some epithelial tumor contexts, reduced BNC2 expression or altered BNC2-related transcripts may be associated with impaired tumor-suppressive or stress-response programs. In contrast, in fibrotic, stromal, or mesenchymal contexts, increased BNC2 activity may promote extracellular matrix remodeling, fibroblast activation, migration, and invasive behavior. Future studies should combine molecular subtyping, lineage-resolved functional experiments, spatial transcriptomics, and clinically annotated cohorts to clarify whether BNC2 acts as a driver, modifier, or biomarker in specific fibrotic and tumor-associated settings.

### 3.6. Context-Dependent Regulatory Effects of BNC2 Across Regenerative, Fibrotic, and Tumor-Associated Settings

Current studies suggest that BNC2 exerts context-dependent effects across epithelial, stromal, regenerative, and fibrotic settings. In epithelial tumor contexts, including high-grade serous ovarian carcinoma, hepatocellular carcinoma, and lung cancer models, reduced BNC2 expression or experimental BNC2 overexpression has been associated with stress-response regulation, interferon-stimulated gene expression, and reduced tumor-cell proliferation [17,18,62]. In contrast, studies of fibrosis, extracellular matrix remodeling, and tumor-associated stromal biology indicate that BNC2 may promote myofibroblastic activation, matrisome gene expression, cell migration, and invasive behavior [15,16]. These divergent findings suggest that BNC2 does not act as a fixed tumor-suppressive or disease-promoting factor but may exert different effects depending on the cellular compartment, including epithelial tumor cells, stromal cells, activated fibroblasts, and tissue-resident progenitor cells.

This context dependence is also evident in tissue repair and skeletal disease. In periosteal stem/progenitor cells, BNC2 supports fracture repair through NuRD-dependent chromatin remodeling and injury-induced progenitor-cell activation [14,16], whereas in fibrotic tissues, it contributes to persistent myofibroblastic activation and extracellular matrix accumulation [15]. Similarly, BNC2-associated regulatory variants have been linked to adolescent idiopathic scoliosis susceptibility, but their relationship with curve progression or disease severity remains less consistent [8,36,53]. These observations suggest that BNC2-related regulatory programs may be beneficial when they support transient progenitor-cell expansion during tissue repair, but pathological when matrix-remodeling or proliferative programs are sustained in fibrotic or tumor-associated microenvironments. Differences in lineage-specific enhancer landscapes, isoform composition, chromatin state, upstream signaling inputs, and inflammatory or mechanical cues may therefore contribute to the divergent effects of BNC2 across disease contexts.

Future studies should move beyond global changes in BNC2 expression and define its direct regulatory programs in specific cellular contexts. Priority should be given to mapping BNC2-binding sites, identifying primary target genes, resolving isoform-specific functions, and distinguishing BNC2 protein function from the effects of BNC2-derived non-coding transcripts such as circBNC2 [49]. Integrating lineage-specific genetic models, single-cell and spatial transcriptomics, chromatin-accessibility profiling, and clinically annotated cohorts will be essential to clarify when BNC2 acts as a regenerative regulator, fibrotic driver, tumor-associated modifier, or disease-susceptibility factor.

## 4. Prospects for Clinical Translation

As research on BNC2 has progressed, its relevance to developmental regulation and disease pathogenesis has gradually become apparent. However, the clinical translation of BNC2-related findings remains at an early stage, because its roles and regulatory mechanisms differ substantially across disease contexts. Current evidence indicates that BNC2 can exert divergent effects in different tissues and cellular environments. In some epithelial tumor contexts, reduced BNC2 expression is associated with impaired stress-response or anti-proliferative programs. In contrast, in fibrosis or stromal remodeling, increased BNC2 activity may promote myofibroblastic activation and pathological extracellular matrix remodeling [15,16,17,18]. This context-dependent pattern suggests that BNC2 cannot be simply classified as a uniformly pathogenic factor, protective regulator, tumor suppressor, or biomarker.

The transcriptional regulation of BNC2 is complex, involving multi-promoter usage, alternative splicing, polyadenylation-site selection, and non-coding enhancer variants [7,19]. Nevertheless, the isoform-specific functions of BNC2, the regulatory elements controlling its tissue-specific expression, and its direct target-gene networks remain incompletely defined. Current studies on BNC2 have mainly focused on genetic associations, expression changes, and model-based functional validation. The upstream regulatory signals, genome-wide binding landscape, downstream transcriptional networks, and interactions with chromatin remodeling, RNA processing, and non-coding RNA regulation still require systematic investigation [6,7,14,49]. These limitations currently constrain both mechanistic interpretation and clinical application.

**Figure 3:**
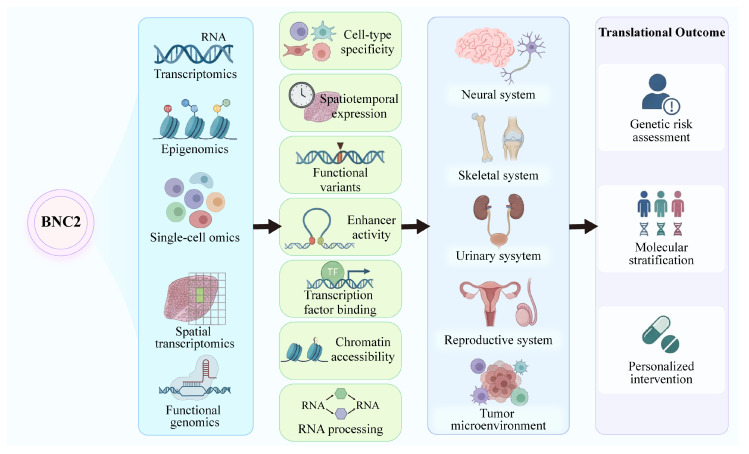
Framework for evaluating the translational potential of BNC2.

Future studies should investigate BNC2 within a multi-layered framework integrating transcriptomics, epigenomics, single-cell multi-omics, spatial transcriptomics, and functional genomics [66,67,68,69]. These approaches may help clarify the cell type-specific expression patterns, spatiotemporal regulatory features, and disease-stage-dependent functions of BNC2. Particular attention should be given to non-coding regulatory variants, transcript heterogeneity, enhancer activity, transcription-factor binding, chromatin accessibility, and RNA-processing-associated mechanisms [6,7,14,18,27,49]. In parallel, tissue-specific and lineage-specific BNC2 intervention models are needed to define its causal roles in the nervous, skeletal, urinary, reproductive, fibrotic, and tumor-associated contexts. Clinically annotated human cohorts will also be essential to determine whether BNC2-related variants, expression changes, or epigenetic signatures can be translated into reliable tools for risk assessment, molecular stratification, or therapeutic decision-making (Figure 3).

The schematic summarizes multi-omics and functional approaches for investigating BNC2-related regulatory mechanisms across neural, skeletal, urinary, reproductive, and tumor-associated contexts. These approaches may support the future evaluation of BNC2-related variants, expression patterns, and epigenetic signatures in genetic risk assessment, molecular stratification, and therapeutic decision-making.

## 5. Conclusions

BNC2 is best understood as a context-dependent nuclear regulatory factor that connects transcriptional regulation, transcript diversity, non-coding regulatory variation, and chromatin remodeling to tissue-specific biological outcomes. Although current evidence links BNC2 to development, tissue repair, fibrosis, cancer susceptibility, pigmentation, reproductive biology, and lower urinary tract development, its direct targets, isoform-specific functions, and disease-relevant regulatory networks remain poorly defined.

Future work should prioritize cell type-resolved functional validation and clinically annotated human cohorts. These studies will be necessary to determine whether BNC2-related variants, expression changes, or epigenetic signatures can be translated into reliable biomarkers, risk-stratification tools, or therapeutic targets.

## Figures and Tables

**Table 1:** Summary of BNC2-related biological contexts and evidence types.

Biological Context/BNC2 Function	Model/Population	Mechanism/Molecular Evidence	Evidence Type	Refs.
Neural energy regulation/feeding suppression	Mouse ARC neurons	LepR-BNC2 neurons, AGRP/NPY circuit	In vivo	[13]
Neuroinflammation/inflammatory and apoptotic suppression	LPS microglia, neuron-like cells	circ-Bnc2/miR-497a-5p/HECTD1	In vitro	[21]
Skeletal development and fracture repair/periosteal activation	Bnc2-KO mice, periosteal progenitors	NuRD/HDAC1/2 remodeling, HOX13	In vivo	[14,30,31,32]
Adolescent idiopathic scoliosis/susceptibility and axial curvature	AIS cohorts, zebrafish assays	rs10738445, YY1, enhancer activity	Clinical + in vivo	[8,33,34,35,36]
Lower urinary tract obstruction/urinary tract development	Affected families, zebrafish models	BNC2 variants, distal outlet obstruction	Clinical + in vivo	[9,37,38]
Male germ-cell development/spermatogonia maintenance and meiosis	Mouse germ cells	BNC1/BNC2, mitotic arrest, meiotic timing	In vivo	[11,32,39]
Epithelial ovarian cancer/susceptibility and stress response	GWAS; fine mapping, HGSOC cells	9p22.2, rs3814113, BRCA1/2, BNC2 methylation	Clinical + in vitro	[10,18,19,20,40,41,42,43]
Pigmentation traits/skin color and pigment pattern	Human cohorts, melanocytes, zebrafish	rs12350739, rs16935073, melanocyte enhancer, BNC2	Clinical + in vitro + in vivo	[12,44,45,46,47,48]
Fibrosis and ECM remodeling/myofibroblast activation	Fibrotic models, fibroblasts	TGF-β/YAP1, collagen, matrisome genes	In vivo + in vitro	[15,16]
Cancer-related BNC2/circBNC2/proliferation, ferroptosis, ECM remodeling	Lung/prostate cancer	IRF7, XAF1, OASs, circBNC2/ACSL6	In vitro + in vivo	[17,49]

## Data Availability

Not applicable.

## References

[1] Razin S.V., Borunova V.V., Maksimenko O.G., Kantidze O.L. (2012). Cys2His2 zinc finger protein family: Classification, functions, and major members. Biochemistry.

[2] Tseng H., Green H. (1992). Basonuclin: A keratinocyte protein with multiple paired zinc fingers. Proc. Natl. Acad. Sci. USA.

[3] Romano R.A., Li H., Tummala R., Maul R., Sinha S. (2004). Identification of Basonuclin2, a DNA-binding zinc-finger protein expressed in germ tissues and skin keratinocytes. Genomics.

[4] Wolfe S.A., Nekludova L., Pabo C.O. (2000). DNA recognition by Cys2His2 zinc finger proteins. Annu. Rev. Biophys. Biomol. Struct..

[5] Vanhoutteghem A., Djian P. (2004). Basonuclin 2: An extremely conserved homolog of the zinc finger protein basonuclin. Proc. Natl. Acad. Sci. USA.

[6] Vanhoutteghem A., Djian P. (2006). Basonuclins 1 and 2, whose genes share a common origin, are proteins with widely different properties and functions. Proc. Natl. Acad. Sci. USA.

[7] Vanhoutteghem A., Djian P. (2007). The human basonuclin 2 gene has the potential to generate nearly 90,000 mRNA isoforms encoding over 2000 different proteins. Genomics.

[8] Ogura Y., Kou I., Miura S., Takahashi A., Xu L., Takeda K., Takahashi Y., Kono K., Kawakami N., Uno K. (2015). A Functional SNP in BNC2 Is Associated with Adolescent Idiopathic Scoliosis. Am. J. Hum. Genet..

[9] Kolvenbach C.M., Dworschak G.C., Frese S., Japp A.S., Schuster P., Wenzlitschke N., Yilmaz Ö., Lopes F.M., Pryalukhin A., Schierbaum L. (2019). Rare Variants in BNC2 Are Implicated in Autosomal-Dominant Congenital Lower Urinary-Tract Obstruction. Am. J. Hum. Genet..

[10] Song H., Ramus S.J., Tyrer J., Bolton K.L., Gentry-Maharaj A., Wozniak E., Anton-Culver H., Chang-Claude J., Cramer D.W., DiCioccio R. (2009). A genome-wide association study identifies a new ovarian cancer susceptibility locus on 9p22.2. Nat. Genet..

[11] Vanhoutteghem A., Messiaen S., Hervé F., Delhomme B., Moison D., Petit J.M., Rouiller-Fabre V., Livera G., Djian P. (2014). The zinc-finger protein basonuclin 2 is required for proper mitotic arrest, prevention of premature meiotic initiation and meiotic progression in mouse male germ cells. Development.

[12] Visser M., Palstra R.J., Kayser M. (2014). Human skin color is influenced by an intergenic DNA polymorphism regulating transcription of the nearby BNC2 pigmentation gene. Hum. Mol. Genet..

[13] Tan H.L., Yin L., Tan Y., Ivanov J., Plucinska K., Ilanges A., Herb B.R., Wang P., Kosse C., Cohen P. (2024). Leptin-activated hypothalamic BNC2 neurons acutely suppress food intake. Nature.

[14] Zhang Z., Zhang L., Jiang B., Chen S., Xing W., Wang P., Lou L., Tang C., Hu X., Suo J. (2026). Basonuclin-2 promotes fracture repair through NuRD-dependent chromatin remodeling in periosteal stem cells. EMBO J..

[15] Bobowski-Gerard M., Boulet C., Zummo F.P., Dubois-Chevalier J., Gheeraert C., Bou Saleh M., Strub J.M., Farce A., Ploton M., Guille L. (2022). Functional genomics uncovers the transcription factor BNC2 as required for myofibroblastic activation in fibrosis. Nat. Commun..

[16] Orang A., Dredge B.K., Liu C.Y., Bracken J.M., Chen C.H., Sourdin L., Whitfield H.J., Lumb R., Boyle S.T., Davis M.J. (2023). Basonuclin-2 regulates extracellular matrix production and degradation. Life Sci. Alliance.

[17] Urgard E., Reigo A., Reinmaa E., Rebane A., Metspalu A. (2017). Human basonuclin 2 up-regulates a cascade set of interferon-stimulated genes with anti-cancerous properties in a lung cancer model. Cancer Cell Int..

[18] Cesaratto L., Grisard E., Coan M., Zandonà L., De Mattia E., Poletto E., Cecchin E., Puglisi F., Canzonieri V., Mucignat M.T. (2016). BNC2 is a putative tumor suppressor gene in high-grade serous ovarian carcinoma and impacts cell survival after oxidative stress. Cell Death Dis..

[19] Buckley M.A., Woods N.T., Tyrer J.P., Mendoza-Fandiño G., Lawrenson K., Hazelett D.J., Najafabadi H.S., Gjyshi A., Carvalho R.S., Lyra P.C. (2019). Functional Analysis and Fine Mapping of the 9p22.2 Ovarian Cancer Susceptibility Locus. Cancer Res..

[20] Winham S.J., Armasu S.M., Cicek M.S., Larson M.C., Cunningham J.M., Kalli K.R., Fridley B.L., Goode E.L. (2014). Genome-wide investigation of regional blood-based DNA methylation adjusted for complete blood counts implicates BNC2 in ovarian cancer. Genet. Epidemiol..

[21] Chen Y., Cao P. (2023). Circ-Bnc2 alleviates neuroinflammation in LPS-stimulated microglial cells to inhibit neuron cell apoptosis through regulating miR-497a-5p/HECTD1 axis. Brain Behav..

[22] Cassandri M., Smirnov A., Novelli F., Pitolli C., Agostini M., Malewicz M., Melino G., Raschellà G. (2017). Zinc-finger proteins in health and disease. Cell Death Discov..

[23] Laity J.H., Lee B.M., Wright P.E. (2001). Zinc finger proteins: New insights into structural and functional diversity. Curr. Opin. Struct. Biol..

[24] Najafabadi H.S., Mnaimneh S., Schmitges F.W., Garton M., Lam K.N., Yang A., Albu M., Weirauch M.T., Radovani E., Kim P.M. (2015). C_2_H_2_ zinc finger proteins greatly expand the human regulatory lexicon. Nat. Biotechnol..

[25] Iuchi S. (2001). Three classes of C_2_H_2_ zinc finger proteins. Cell. Mol. Life Sci..

[26] Zhang X., Blumenthal R.M., Cheng X. (2024). Updated understanding of the protein-DNA recognition code used by C_2_H_2_ zinc finger proteins. Curr. Opin. Struct. Biol..

[27] Nabeel-Shah S., Pu S., Burns J.D., Braunschweig U., Ahmed N., Burke G.L., Lee H., Radovani E., Zhong G., Tang H. (2024). C2H2-zinc-finger transcription factors bind RNA and function in diverse post-transcriptional regulatory processes. Mol. Cell.

[28] Iuchi S., Green H. (1999). Basonuclin, a zinc finger protein of keratinocytes and reproductive germ cells, binds to the rRNA gene promoter. Proc. Natl. Acad. Sci. USA.

[29] Tian Q., Kopf G.S., Brown R.S., Tseng H. (2001). Function of basonuclin in increasing transcription of the ribosomal RNA genes during mouse oogenesis. Development.

[30] Vanhoutteghem A., Maciejewski-Duval A., Bouche C., Delhomme B., Hervé F., Daubigney F., Soubigou G., Araki M., Araki K., Yamamura K. (2009). Basonuclin 2 has a function in the multiplication of embryonic craniofacial mesenchymal cells and is orthologous to disco proteins. Proc. Natl. Acad. Sci. USA.

[31] Yamamoto S., Uchida Y., Ohtani T., Nozaki E., Yin C., Gotoh Y., Yakushiji-Kaminatsui N., Higashiyama T., Suzuki T., Takemoto T. (2019). Hoxa13 regulates expression of common Hox target genes involved in cartilage development to coordinate the expansion of the autopodal anlage. Dev. Growth Differ..

[32] Vanhoutteghem A., Delhomme B., Hervé F., Nondier I., Petit J.M., Araki M., Araki K., Djian P. (2016). The importance of basonuclin 2 in adult mice and its relation to basonuclin 1. Mech. Dev..

[33] Ogura Y., Takeda K., Kou I., Khanshour A., Grauers A., Zhou H., Liu G., Fan Y.H., Zhou T., Wu Z. (2018). An international meta-analysis confirms the association of BNC2 with adolescent idiopathic scoliosis. Sci. Rep..

[34] Xu L., Xia C., Qin X., Sun W., Tang N.L., Qiu Y., Cheng J.C., Zhu Z. (2017). Genetic variant of BNC2 gene is functionally associated with adolescent idiopathic scoliosis in Chinese population. Mol. Genet. Genom..

[35] Man G.C., Tang N.L., Chan T.F., Lam T.P., Li J.W., Ng B.K., Zhu Z., Qiu Y., Cheng J.C. (2019). Replication Study for the Association of GWAS-associated Loci with Adolescent Idiopathic Scoliosis Susceptibility and Curve Progression in a Chinese Population. Spine.

[36] Wang W., Chen T., Liu Y., Wang S., Yang N., Luo M. (2022). Predictive value of single-nucleotide polymorphisms in curve progression of adolescent idiopathic scoliosis. Eur. Spine J..

[37] Bhoj E.J., Ramos P., Baker L.A., Garg V., Cost N., Nordenskjöld A., Elder F.F., Bleyl S.B., Bowles N.E., Arrington C.B. (2011). Human balanced translocation and mouse gene inactivation implicate Basonuclin 2 in distal urethral development. Eur. J. Hum. Genet..

[38] Kolvenbach C.M., Dworschak G.C., Rieke J.M., Woolf A.S., Reutter H., Odermatt B., Hilger A.C. (2023). Modelling human lower urinary tract malformations in zebrafish. Mol. Cell. Pediatr..

[39] Shimada R., Ishiguro K.I. (2023). Cell cycle regulation for meiosis in mammalian germ cells. J. Reprod. Dev..

[40] Labidi-Galy S.I., Papp E., Hallberg D., Niknafs N., Adleff V., Noe M., Bhattacharya R., Novak M., Jones S., Phallen J. (2017). High grade serous ovarian carcinomas originate in the fallopian tube. Nat. Commun..

[41] Perets R., Wyant G.A., Muto K.W., Bijron J.G., Poole B.B., Chin K.T., Chen J.Y., Ohman A.W., Stepule C.D., Kwak S. (2013). Transformation of the fallopian tube secretory epithelium leads to high-grade serous ovarian cancer in Brca; Tp53; Pten models. Cancer Cell.

[42] Ramus S.J., Kartsonaki C., Gayther S.A., Pharoah P.D., Sinilnikova O.M., Beesley J., Chen X., McGuffog L., Healey S., Couch F.J. (2011). Genetic variation at 9p22.2 and ovarian cancer risk for BRCA1 and BRCA2 mutation carriers. J. Natl. Cancer Inst..

[43] Vigorito E., Kuchenbaecker K.B., Beesley J., Adlard J., Agnarsson B.A., Andrulis I.L., Arun B.K., Barjhoux L., Belotti M., Benitez J. (2016). Fine-Scale Mapping at 9p22.2 Identifies Candidate Causal Variants That Modify Ovarian Cancer Risk in BRCA1 and BRCA2 Mutation Carriers. PLoS ONE.

[44] Jacobs L.C., Wollstein A., Lao O., Hofman A., Klaver C.C., Uitterlinden A.G., Nijsten T., Kayser M., Liu F. (2013). Comprehensive candidate gene study highlights UGT1A and BNC2 as new genes determining continuous skin color variation in Europeans. Hum. Genet..

[45] Hider J.L., Gittelman R.M., Shah T., Edwards M., Rosenbloom A., Akey J.M., Parra E.J. (2013). Exploring signatures of positive selection in pigmentation candidate genes in populations of East Asian ancestry. BMC Evol. Biol..

[46] Kim Y., Yin J., Huang H., Jorgenson E., Choquet H., Asgari M.M. (2022). Genome-wide association study of actinic keratosis identifies new susceptibility loci implicated in pigmentation and immune regulation pathways. Commun. Biol..

[47] Seo J.Y., You S.W., Shin J.G., Kim Y., Park S.G., Won H.H., Kang N.G. (2022). GWAS Identifies Multiple Genetic Loci for Skin Color in Korean Women. J. Investig. Dermatol..

[48] Eriksson N., Macpherson J.M., Tung J.Y., Hon L.S., Naughton B., Saxonov S., Avey L., Wojcicki A., Pe’er I., Mountain J. (2010). Web-based, participant-driven studies yield novel genetic associations for common traits. PLoS Genet..

[49] Pan X., Chen K., Gao W., Xu M., Meng F., Wu M., Wang Z.Q., Li Y.Q., Xu W., Zhang M. (2025). Circular RNA circBNC2 inhibits tumorigenesis by modulating ferroptosis and acts as a nanotherapeutic target in prostate cancer. Mol. Cancer.

[50] Wright C.M., Schneider S., Smith-Edwards K.M., Mafra F., Leembruggen A.J.L., Gonzalez M.V., Kothakapa D.R., Anderson J.B., Maguire B.A., Gao T. (2021). scRNA-Seq Reveals New Enteric Nervous System Roles for GDNF, NRTN, and TBX3. Cell. Mol. Gastroenterol. Hepatol..

[51] Weinsheimer S., Bendjilali N., Nelson J., Guo D.E., Zaroff J.G., Sidney S., McCulloch C.E., Al-Shahi Salman R., Berg J.N., Koeleman B.P. (2016). Genome-wide association study of sporadic brain arteriovenous malformations. J. Neurol. Neurosurg. Psychiatry.

[52] Makki N., Zhao J., Liu Z., Eckalbar W.L., Ushiki A., Khanshour A.M., Wu J., Rios J., Gray R.S., Wise C.A. (2021). Genomic characterization of the adolescent idiopathic scoliosis-associated transcriptome and regulome. Hum. Mol. Genet..

[53] Terhune E., Heyn P., Piper C., Wethey C., Monley A., Cuevas M., Hadley Miller N. (2024). Association between genetic polymorphisms and risk of adolescent idiopathic scoliosis in case-control studies: A systematic review. J. Med. Genet..

[54] Tuncay I.O., Lee E.K., Gustafson A., Lee Y., Jung D., Koh J.Y., Lee W., Lee S., Shazand K. (2025). Whole genome sequencing in adolescent idiopathic scoliosis cohort implicates multiple biological pathways. npj Genom. Med..

[55] Lissauer D., Morris R.K., Kilby M.D. (2007). Fetal lower urinary tract obstruction. Semin. Fetal Neonatal Med..

[56] Roberts N.A., Chan M.M.Y., Woolf A.S. (2025). The biology of congenital urinary bladder outflow obstruction. Trends Mol. Med..

[57] Mustafa H.J., Khalil A., Johnson S., Gordijn S.J., Ganzevoort W., Melling C., Koh C.J., Mandy G.T., Kilby M.D., Johnson A. (2024). Fetal lower urinary tract obstruction: International Delphi consensus on management and core outcome set. Ultrasound Obs. Gynecol..

[58] Robertson W.B., Hayes J.A. (1969). Congenital diaphragmatic obstruction of the male posterior urethra. Br. J. Urol..

[59] Del Bino S., Duval C., Bernerd F. (2018). Clinical and Biological Characterization of Skin Pigmentation Diversity and Its Consequences on UV Impact. Int. J. Mol. Sci..

[60] Jacobs L.C., Hamer M.A., Gunn D.A., Deelen J., Lall J.S., van Heemst D., Uh H.W., Hofman A., Uitterlinden A.G., Griffiths C.E.M. (2015). A Genome-Wide Association Study Identifies the Skin Color Genes IRF4, MC1R, ASIP, and BNC2 Influencing Facial Pigmented Spots. J. Investig. Dermatol..

[61] Lang M.R., Patterson L.B., Gordon T.N., Johnson S.L., Parichy D.M. (2009). Basonuclin-2 requirements for zebrafish adult pigment pattern development and female fertility. PLoS Genet..

[62] Wu Y., Zhang X., Liu Y., Lu F., Chen X. (2016). Decreased Expression of BNC1 and BNC2 Is Associated with Genetic or Epigenetic Regulation in Hepatocellular Carcinoma. Int. J. Mol. Sci..

[63] Yang M., Arai E., Takahashi Y., Totsuka H., Chiku S., Taniguchi H., Katai H., Sakamoto H., Yoshida T., Kanai Y. (2020). Cooperative participation of epigenomic and genomic alterations in the clinicopathological diversity of gastric adenocarcinomas: Significance of cell adhesion and epithelial-mesenchymal transition-related signaling pathways. Carcinogenesis.

[64] Nation J.B., Cabot-Miller J., Segal O., Lucito R., Adaricheva K. (2021). Combining Algorithms to Find Signatures That Predict Risk in Early-Stage Stomach Cancer. J. Comput. Biol..

[65] Zhang Z., Liang Z., Li D., Wang L., Chen Y., Liang Y., Jiao W., Niu H. (2022). Development of a CAFs-related gene signature to predict survival and drug response in bladder cancer. Hum. Cell.

[66] Zhang L., Chen D., Song D., Liu X., Zhang Y., Xu X., Wang X. (2022). Clinical and translational values of spatial transcriptomics. Signal Transduct. Target. Ther..

[67] Grandi F.C., Modi H., Kampman L., Corces M.R. (2022). Chromatin accessibility profiling by ATAC-seq. Nat. Protoc..

[68] Jain S., Eadon M.T. (2024). Spatial transcriptomics in health and disease. Nat. Rev. Nephrol..

[69] Li K., Ouyang M., Zhan J., Tian R. (2023). CRISPR-based functional genomics screening in human-pluripotent-stem-cell-derived cell types. Cell Genom..

